# The Impact of Autism Spectrum Disorder on Parents in Arab Countries: A Systematic Literature Review

**DOI:** 10.3389/fpsyg.2022.955442

**Published:** 2022-07-14

**Authors:** Jamal M. Alkhateeb, Muna S. Hadidi, Wissam Mounzer

**Affiliations:** ^1^Department of Special Education, The University of Jordan, Amman, Jordan; ^2^Department of Special Education, Stockholm University, Stockholm, Sweden

**Keywords:** autism spectrum disorder, Arab countries, parents, caregivers, stress, burden, wellbeing, quality of life

## Abstract

**Background:**

Having a child with autism spectrum disorder can have significant psychological effects on parents. This systematic review summarizes the current state of literature underscoring the impact of autism spectrum disorder (ASD) on parents in Arab countries

**Methods:**

A systematic search of seven databases (PubMed, Scopus, ProQuest, Google Scholar, ERIC, Academic Search Complete, and PsycINFO) was performed, which identified 24 studies (20 quantitative studies and four qualitative studies) that included 3,299 parents or caregivers of children with ASD. These studies were conducted in 10 Arab countries (Saudi Arabia, Jordan, Egypt, Kuwait, Bahrain, Oman, Emirates, Palestine, Qatar, and Lebanon).

**Results:**

The majority of the included studies found that ASD has a significant negative impact on the mental health and wellbeing of Arab parents. It was found that parents of children with ASD have a poor quality of life (QoL) and an increased risk of psychological disorders. These findings were in contrast to findings of parents of typically developing children and children with other developmental disorders. Challenges faced by parents of children with ASD were associated with several child- and parent-related factors. The most common coping strategy used by parents was religious coping.

**Conclusion:**

The impact of ASD on parents has only recently gained traction among researchers in Arab countries. Despite several knowledge gaps, published studies have provided useful information outlining the impact of ASD on parents in some of these countries. Further research comprising larger random samples and using varied research and data-collection methods is required to understand the multifaceted challenges experienced by parents raising children with ASD in Arab countries.

## Introduction

Autism spectrum disorder (ASD) is a lifelong neurodevelopmental disorder that intersects racial, ethnic, and socioeconomic boundaries, and is characterized by persistent impairments in social interactions, verbal and non-verbal communication, as well as restricted and repetitive patterns of behavior, interest, or activities (American Psychiatric Association, 2013). Over the past three decades, there has been a global increase in the prevalence of ASD (Elsabbagh et al., 2012; Blaxill et al., 2022).

Extensive research indicates that parents of children with ASD often experience elevated levels of parenting stress (Hoffman et al., 2009), increased mental health problems (Cohrs and Leslie, 2017), and reduced physical health (Smith et al., 2012; Eisenhower et al., 2013) in contrast to parents of typically developing children (Ingersoll and Hambrick, 2011; Padden and James, 2017), and parents of children diagnosed with other disabilities (Hayes and Watson, 2013; Barroso et al., 2018). Furthermore, ASD significantly impacts family life as well as the marriage system (Karst and Van Hecke, 2012; Serrata, 2012; Pisula and Porebowicz-Dörsmann, 2017).

Increasing evidence suggests that the impact of ASD on parents may be attributed to the severity of the emotional and behavioral problems exhibited by the child (Leyfer et al., 2006; Baker et al., 2012; Karst and Van Hecke, 2012). Furthermore, this is exacerbated by exhaustive caregiving demands, poor parental coping capabilities, lack of support (Weiss et al., 2014; Papadopoulos, 2021), economic burden (Ou et al., 2015), as well as the perception and understanding of ASD among parents (Ilias et al., 2018).

The literature also suggests that cultural background is an important variable to consider when analyzing the impact of ASD on parents (Ilias et al., 2018; Zakirova-Engstrand et al., 2020). Parents' awareness and beliefs about the etiology and prognosis of ASD can affect parental responses, coping strategies, as well as treatment decision-making (Hebert and Koulouglioti, 2010; Zuckerman et al., 2016; Brewton et al., 2021).

The majority of ASD research has been conducted across the United States (US) and the United Kingdom (UK) (Clark and Adams, 2020; Roche et al., 2021), in contrast to other parts of the world where research on ASD is relatively limited (Samadi and McConkey, 2011; Rice et al., 2012). In Arab countries, ASD is a new field of research that has gradually evolved in the past two decades. However, severe information disparities with regards to the different aspects of ASD have been noted, namely, epidemiology, characteristics, burden, as well as support available to children and families.

To date, four literature reviews have been published on ASD research in Arab countries (Hussein and Taha, 2013; Salhia et al., 2014; Alnemary et al., 2017; Alallawi et al., 2020). Alallawi et al. (2020) conducted a systematic scoping review of social, educational, and psychological research relevant to persons with ASD and their families in Arab countries. Using eight databases, Alallawi et al. (2020) identified 70 studies published predominantly by researchers from Saudi Arabia and Lebanon. Most of the identified studies investigated the prevalence of ASD; diagnosis issues; the experiences and outcomes of Arab caregivers for individuals with ASD; as well as the social and communication behavior of Arab individuals with ASD. The results of the scoping review revealed significant gaps in research related to ASD interventions and services. Furthermore, upon appraisal of the identified studies, the authors found them to be of low quality.

Alnemary et al. (2017) reviewed published research on ASD in the Arab world from 1992 to 2014. The authors searched for studies published in English using PubMed, Web of Science, and EMBASE databases. In total, the authors identified 142 publications that were produced mostly by researchers in Saudi Arabia, Egypt, and Oman. For the most part, these publications addressed the biology, risk factors, and diagnosis of ASD. However, limited studies investigated intervention, services, infrastructure and surveillance, or life span issues related to ASD.

Hussein and Taha (2013) analyzed published literature on ASD in the Arab world from 1992 to 2012 using the Medline database. In total, 79 articles were identified that focused predominantly on the etiology of ASD as opposed to services and interventions.

Finally, Salhia et al. (2014) conducted a systemic review of the epidemiology of ASD in Arab Gulf countries, namely, Saudi Arabia, United Arab Emirates, Oman, Kuwait, Qatar, and Bahrain. The literature search was conducted using PubMed and ScienceDirect databases. However, limited studies investigating the epidemiology of ASD were identified by the authors. Those studies showed a prevalence rate ranging from 1.4 to 29 per 10,000 persons. Furthermore, no studies explored the burden of ASD on the child, family, or society.

Due to the limited scientific evidence available on challenges faced by parents raising children with ASD in Arab countries (Alnemary et al., 2017; Al Khateeb et al., 2019; Alallawi et al., 2020), the current systematic literature review was undertaken. This review aimed to address this crucial knowledge gap in ASD research by locating and synthesizing all studies underscoring the impact of ASD on parents in Arab countries.

## Methods

### Search Strategy

Studies investigating the impact of ASD on parents in Arab countries were reviewed and analyzed. To identify the relevant literature, seven databases were searched, namely, PubMed, Google Scholar, ERIC, CINAHL, Education Research Complete, Springer Link, and Psychology and Behavioral Sciences Collection. The review was conducted and reported according to the Preferred Reporting Items for Systematic Reviews and Meta-Analyses (PRISMA; Moher et al., 2009). Three sets of search terms were used in the initial search, namely, “Autism” OR “ASD” OR “Asperger” OR “Pervasive Developmental Disorders” AND “parents” OR “mothers” OR “fathers” OR “stress” OR “well-being” OR “mental health problems” AND “Arab countries” OR “Algeria” OR “Egypt” OR “Libya” OR “Tunisia” OR “Morocco” OR “Mauritania” OR “Sudan” OR “Somalia” OR “Djibouti” OR “Bahrain” OR “Emirates” OR “Oman” OR “Kuwait” OR “Qatar” OR “Saudi” OR “Yemen” OR “Jordan” OR “Syria” OR “Iraq” OR “Lebanon” OR “Comoros” OR “Palestine.” Furthermore, the key concept of ASD was searched using the term “Arab countries OR Arab World” to identify studies that might have been omitted. Finally, the reference lists of the studies included in the review were scanned for additional studies.

### Inclusion and Exclusion Criteria

This review included studies that were: empirical, conducted in an Arab country, related to ASD impact on parents, published in English, and published in a peer-reviewed journal. Literature reviews, doctoral dissertations or master theses, conference papers, chapters, and theoretical articles were excluded. Publication dates were not restricted to ensure all possibly relevant articles were included.

### Study Selection

**Figure 1:**
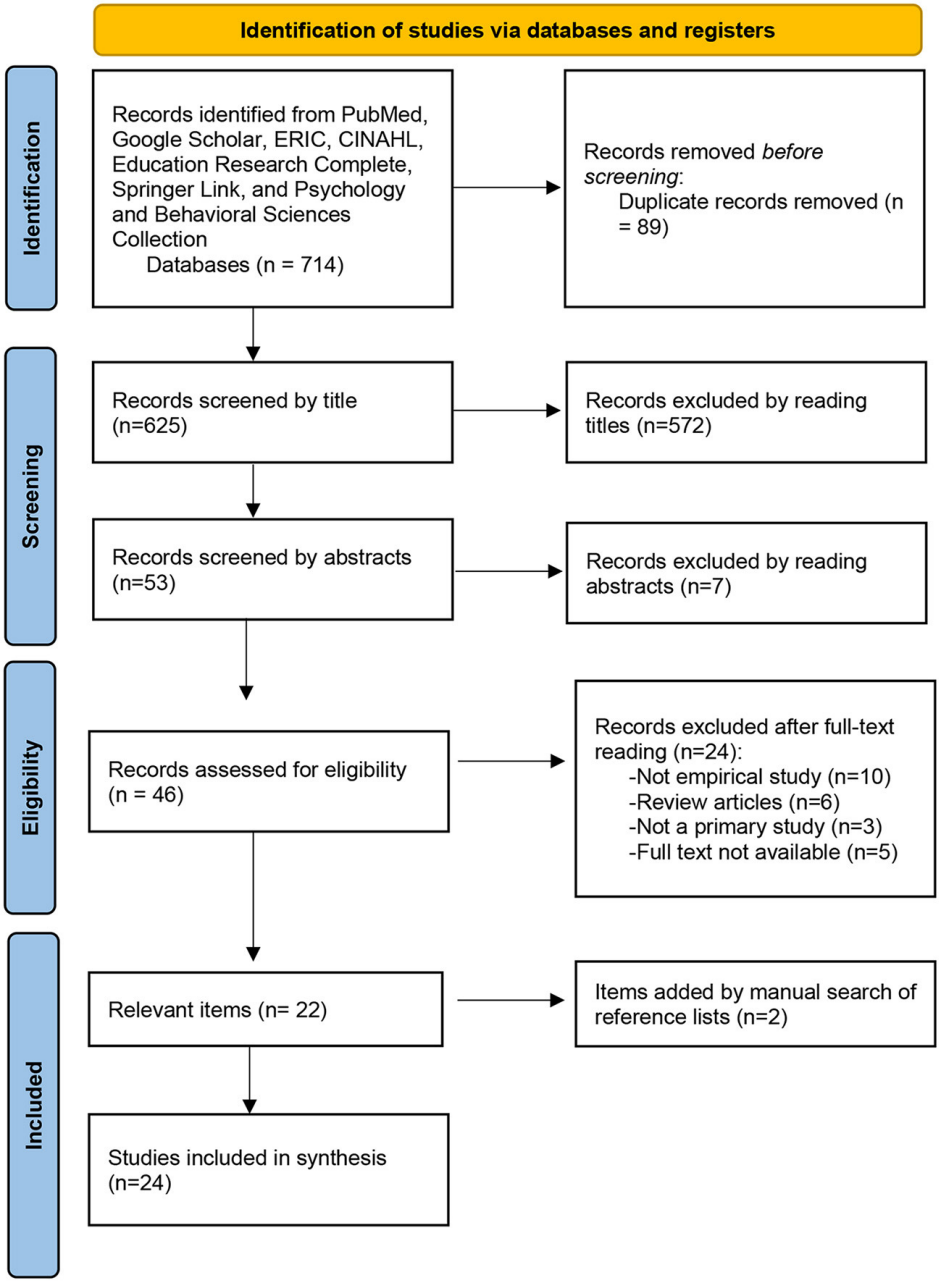
PRISMA flow chart of the study identification process.

Figure 1 presents a flowchart of the process used to identify and select relevant studies. The database and keyword search listed above yielded 714 studies. Eighty-eight articles were excluded due to duplication across the databases. Upon screening the titles for eligibility, 572 articles were excluded. Furthermore, seven articles were excluded after reading the abstracts. After assessing the full texts of the remaining 47 articles, 22 articles were excluded. These articles were excluded because 10 of them were not empirical studies, six were review articles, three articles were not available in full text and five articles were not primary studies. A manual search of reference lists of the identified articles resulted in the identification of two additional studies. Therefore, the final sample included 24 studies.

### Charting the Data

The authors developed a coding form to assist with the data extraction. The coding form comprised sections designed to obtain information regarding the author(s), publication year, country, purpose, methods, and key findings. Thereafter, the first author used this chart to extract data from the included articles. The second author independently extracted data for 29% (*n* = 10) of the included articles. Disagreements among the authors were resolved through discussion. Finally, the information was analyzed to identify themes and gaps in the literature and to suggest implications for future research and practice.

## Results

### Description of the Included Studies

Table 1 summarizes the study design, sample, main objectives, methods, and key findings of the 24 included publications. The studies underscoring the impact of ASD on parents in the Arab world were conducted in 10 countries. Among these publications, six were from Saudi Arabia, six were from Jordan, three were from Egypt, two were from Kuwait, two were from Bahrain, and one study was from Oman, Emirates, Palestine, Qatar, and Lebanon. The remaining Arab countries, namely, Algeria, Libya, Tunisia, Morocco, Mauritania, Sudan, Somalia, Djibouti, Yemen, Syria, Iraq, or Comoros, did not have any published articles.

**Table 1:** Overview of the included studies (*n* = 24).

**Author (year), country**	**Sample**	**Study design**	**Purpose**	**Data collection method(s)**	**Key findings**
Ahmad and Dardas (2015), Jordan	101 fathers of children with ASD	Quantitative cross-sectional study	Identify variables that can potentially predict the psychological health of fathers of children with ASD	World Health Organization Quality of Life Assessment (WHOQOL)	• Fathers of children with ASD reported high levels of stress
					• Fathers' personal characteristics (parent distress and fathers' level of education) and characteristics of their children (difficult child characteristics and child's gender) significantly affected fathers' quality of life (QoL)
Al Ansari et al. (2021), Bahrain	126 mothers of children with ASD, 43 mothers of children with diabetes mellitus (DM), and 116 mothers of typically developing children	Quantitative case-control study	Compare the prevalence of symptoms of depression, anxiety, and stress among mothers of children with (ASD), type 1 DM, and typical development	Depression, Anxiety and Stress Scale (DASS-21) and Perceived Stress Scale (PSS).	• Mothers of children with ASD or DM had higher levels of depression, anxiety, and stress than mothers of typically developing children
					• Mothers of children with ASD reported higher levels of depression, whereas mothers of children with DM had higher anxiety and stress than mothers of the control group
Al-Ansari and Jahrami (2018), Bahrain	30 mothers of children with ASD, 30 mothers of children with intellectual disability (ID), and 30 mothers of children without disabilities	Quantitative cross-sectional study	Evaluate physical health, mental health, and the QoL of mothers of ASD and ID children compared to mothers of typically developing children	Face-to-face interviews with mothers.	• Mothers of children with ASD reported more psychological and environmental problems than mothers of ID and children without disabilities
					• All groups obtained a similar score on the QoL total score and physical health score
Alenazi et al. (2020), Saudi Arabia	84 parents of children with ASD	Quantitative cross-sectional study	Evaluate the effect of ASD on the QoL of parents	36-Item Short Form Survey (SE-36)	• A high percentage of parents of children with ASD had impaired QoL
					• Main domains affected were role limitations as a result of emotional problems, energy/fatigue, and social functioning
Al-Farsi et al. (2016), Oman	220 parents of children with ASD, 109 parents of children with ID, and 125 parents of typically developing children	Quantitative case–control study	Compare stress, anxiety, and depression among parents of children with and without ASD	DASS-21	• Parents of children with ASD had higher levels of stress, depression, and anxiety than parents of children with ID or parents of typically developing children
Al-Kandari et al. (2017), Kuwait	198 mothers of children with ASD	Quantitative cross-sectional study	Investigate coping strategies of mothers of children with ASD	Brief version of the Coping Orientation to Problems Experienced Inventory (Brief-COPE)	• The majority of mothers of children with ASD reported decreased ability to perform social duties, take care of themselves or enjoy life
					• Strain suffered by mothers was inversely associated with maternal education
					• There was a significant association between the mothers' ability to enjoy life and receiving support from the family and support groups
					• Religion, acceptance, and positive reframing were the three most common coping strategies
Almansour et al. (2013), Saudi Arabia	50 parents of children with ASD 50 controls	Quantitative retrospective cohort study	Compare depression and anxiety in parents of children with ASD and parents of normally developing children, and determine factors associated with depression and anxiety among parents of children with ASD	A self-report questionnaire, Hospital Anxiety and Depression (HAD) Scale	• Depression and anxiety levels were significantly higher among cases compared with controls
					• Anxiety level was higher among more educated parents and with number of children with ASD in the family
Al-Masa'deh et al. (2020), Jordan	223 parents (122 fathers and 101 mothers) of children with ASD	Quantitative survey study	Identify daily social and emotional challenges encountered by parents of children with ASD	A scale developed by researchers to assess social and emotional challenges encountered by parents	• Most common social challenges were lack of social support, and most common emotional challenges were anger and aggression
					• Social and emotional burden was associated with severity of ASD, household income, and child gender
Alnazly and Abojedi (2019), Jordan	123 parents of children with ASD	Quantitative cross-sectional study	Investigate psychological distress among parents of children with ASD	Oberst Caregiver Burden Scale time and difficulty subscales (OCBS-T and OCBS-D), Bakas Caregiving Outcome Scale (OCBS), and Hospital Anxiety and Depression Scale (HADS)	• Parents experienced moderate levels of burden, negative life changes, and borderline depression and anxiety
					• Parents' burden was related to their marital status, employment, age, and the number of female family members
Al-qahtani (2018), Saudi Arabia	157 caregivers of children with ASD	Quantitative cross-sectional study	Assess burden experienced by family members of children with ASD	Zarit Burden Interview	• Social burden was the most common among caregivers, followed by physical, financial and lastly psychological burden
					• Higher financial and psychological burdens were reported by older caregivers, and higher physical burden was experienced by caregivers with lower levels of education
Alshahrani and Algashmari (2021), Saudi Arabia	50 parents of children with ASD (30 fathers and 20 mothers)	Quantitative cross-sectional study	Explore extent of anxiety and depressive disorders in parents of children with ASD	Patient Health Questionnaire (PHQ)*-*9	• The vast majority of parents had mild to moderate depression
					• Depressive symptoms were significantly associated with severity of ASD but were not associated with economic status
Alshekaili et al. (2019), Saudi Arabia	92 parents/caregivers of children with ASD	Quantitative cross-sectional study	Examine prevalence of depressive symptoms among parents/caregivers of children with ASD, and investigate the clinical and sociodemographic correlates of depressive symptoms	(PHQ)-9	• 71% of parents/caregivers of children with ASD had depressive symptoms
					• Unemployment and being a single parent/caregiver in the family were both significant correlates of depressive symptoms
Amireh (2019), Egypt	55 parents of children with ASD, 28 parents of children with Down's syndrome, and 88 parents of typically developing children	Quantitative cross-sectional study	Evaluate levels of stress among parents of children with ASD and children with Down's syndrome compared with parents of typically developing children, and identified coping strategies used by parents	Parenting Stress Index—Short Form (PSI–SF).	• Parents of children with ASD experienced the highest level of stress when compared to parents of Down syndrome and typically developing children
					• Religious coping was the most commonly used strategy by parents
Dababnah and Parish (2013), West Bank	24 parents (20 mothers and four fathers) of children with ASD	Qualitative interview and focus group study	Investigate the experiences of parents raising children with ASD	Interviews and focus groups	• Depression was pervasive among parents
					• Parents demonstrated remarkable resilience despite experiencing significant psychological, emotional and financial stress
					• Negative family and community attitudes were a tremendous source of stress for parents
					• Some parents used religious coping or withdrawal from community and denial of diagnosis, while others aimed to increase social interactions and access information
Dardas and Ahmad (2014), Jordan	184 parents (70 fathers and 114 mothers) of children with ASD	Quantitative cross-sectional study	Investigate QoL between of parents of children with ASD	WHOQOL-BREF, Ways of Coping Checklist-Revised (WCC-R), Ways of Coping Checklist-Revised (WCC-R), PSI-SF	• Children with ASD had a significant impact on their parents' QoL and wellbeing
					• No significant differences were found between fathers and mothers in parental stress or QoL and wellbeing •QoL levels were significantly associated with parenting stress, coping strategies, and demographic characteristics
El-Monshed and Amr (2021), Egypt	94 mothers of children with ASD	Quantitative cross-sectional study	Assess perceived stress among mothers of children with ASD	Perceived Stress Scale (PSS).	• Mothers of children with ASD experienced high levels of stress
					• Residence, marital status, educational level, financial status, and family history of psychiatric illnesses had a statistically significant relation with mothers' perceived stress
Fido and Al Saad (2013), Kuwait	120 parents of children with ASD and 125v parents of typically developing children	Quantitative cross-sectional study	Evaluate the prevalence of parental depression in families of children with ASD	Beck's Depression Inventory (BDI).	• Mothers of children with ASD reported a significantly higher levels of depression symptoms than mothers of typically developing children
					• No significant differences were found between fathers of children with ASD and fathers of typically developing children
					• Single mothers in both groups had higher elevated depression scores than mothers living with partners
Gobrial (2018), Egypt	14 mothers of children with ASD	Qualitative grounded theory	Investigate experiences of mothers caring for children with ASD	Semi-structured interviews.	• ASD had a significant impact on the social life and emotional wellbeing of mothers
					• Inadequate education, healthcare and stigma constituted the main issues for mothers
Kheir et al. (2012), Qatar	98 caregivers (56 caregivers of children with ASD and 42 caregivers of typically developing children)	Quantitative cross-sectional study	Assess the QoL of caregivers of children with ASD	Standard Recall Short Form 36 (SF-36 v2)	• General health component of the QoL scale used was significantly poorer in caregivers of children with ASD
Khusaifan and El Keshky (2021), Saudi Arabia	131 parents of children with ASD	Quantitative cross-sectional study	Assess impact of social support as a mediator and/or a moderator between parental stress and life satisfaction among parents of children with ASD	Family Stress and Coping Interview-Adapted Scale (FSCI-A), the Satisfaction with Life Scale (SWLS), and the Multidimensional Scale of Perceived Social Support Scale (MSPSS)	• Parents of children with ASD had a high level of stress and stress-related conditions
					• Social support had a significant role in reducing parental stress
Lamba et al. (2022), Emirates	17 mothers of children with ASD	Qualitative In-depth semi-structured interview study	Explore challenges and support structures of mothers with children with ASD	Interviews	• Majority of mothers were extremely satisfied with support groups
					• Several mothers, however, were rejected by extended family members and faced hardships raising their children
Obeid and Daou (2015), Lebanon	163 mothers (65 mothers of children with ASD and 98 mothers of children of typical development)	Quantitative cross-sectional study	Determine the predictors of wellbeing in mothers of children with ASD	Brief COPE scale, Interpersonal Support Evaluation List (ISEL), Indian Scale for Assessment of ASD (ISAA), and General Health Questionnaire (GHQ-12)	• Mothers of children with ASD had significantly lower wellbeing than mothers of typically developing children
					• A significant correlation was found between child's behavioral problems and maternal wellbeing •Mother of children with ASD showed lower levels of perceived social support
Rayan and Ahmad (2017), Jordan	187 parents of children with ASD	Quantitative descriptive correlational study	Examine association between positive reappraisal coping (PRC) and psychological distress in parents of children with ASD	DASS-21, and Positive Reappraisal Coping (PRC) Subscale of the Cognitive Emotion Regulation Questionnaire (CERQ)	• 80, 86, and 82% of parents had higher than normal levels of depression, anxiety and stress, respectively
					• PRC was found to be a stronger predictor of psychological distress in parents than parental age or gender
Shattnawi et al. (2021), Jordan	14 mothers of children with ASD	Qualitative phenomenological study	Explore experiences of mothers caring for a child with ASD	Semi-structured interviews.	• All mothers experienced physical, psychological, financial, and social burdens.

Twenty publications (84%) were quantitative studies, which were mostly cross-sectional, while four publications (16%) were qualitative studies. The samples used in eight of the studies included only mothers (Obeid and Daou, 2015; Al-Kandari et al., 2017; Al-Ansari and Jahrami, 2018; Gobrial, 2018; Al Ansari et al., 2021; El-Monshed and Amr, 2021; Shattnawi et al., 2021; Lamba et al., 2022), while one study (Ahmad and Dardas, 2015) included only fathers.

### Data Collection Tools

This review employed 19 different data collection tools including the World Health Organization Quality of Life Assessment (WHOQOL-BREF), the Depression, Anxiety and Stress Scale (DASS-21), the Perceived Stress Scale (PSS), the 36-Item Short Form Survey (SE-36), the Brief Version of the Coping Orientation to Problems Experienced Inventory (Brief -COPE), the Hospital Anxiety and Depression Scale (HADS), Oberst Caregiver Burden Scale time and difficulty subscales (OCBS-T and OCBS-D), Bakas Caregiving Outcome Scale (OCBS), Zarit Burden Interview (ZBI-12), Patient Health Questionnaire (PHQ)-9, Parenting Stress Index—Short Form (PSI—SF), Ways of Coping Checklist-Revised (WCC-R), Beck's Depression Inventory (BDI), the Standard Recall Short Form 36 (SF-36 v2), Family Stress and Coping Interview-Adapted Scale (FSCI-A), the Satisfaction with Life Scale (SWLS), Interpersonal Support Evaluation List (ISEL), General Health Questionnaire (GHQ-12), and the Positive Reappraisal Coping (PRC) Subscale of the Cognitive Emotion Regulation Questionnaire (CERQ).

The included studies were all published in the past 10 years and most of these studies (*n* = 16, 67%) were conducted between 2017 and 2021.

### Participants in the Included Studies

In total, 3,299 parents or caregivers, namely, 2,415 parents or caregivers of children with ASD and 884 controls, were included as participants in these studies. The total number of mothers and fathers of children with ASD was 813 and 327, respectively. Eleven studies comprising 1,275 parents or caregivers of children with ASD did not specify the gender of the parent and referred only to the term “parents” (Kheir et al., 2012; Almansour et al., 2013; Fido and Al Saad, 2013; Al-Farsi et al., 2016; and Rayan and Ahmad, 2017; Al-qahtani, 2018; Alnazly and Abojedi, 2019; Alshekaili et al., 2019; Amireh, 2019; Alenazi et al., 2020; Khusaifan and El Keshky, 2021). All the studies used non-probability sampling methods (i.e., convenience samples, purposefully selected samples, snowball samples, and voluntary samples) except for one study which used a probability sample (a systematic random sample; Alshekaili et al., 2019).

### Impact of ASD on Parents

Data from studies included in this review revealed that parents raising children with ASD in Arab countries experience considerable stress and strain. Nine studies reported high levels of parental stress (Dababnah and Parish, 2013; Dardas and Ahmad, 2014; Ahmad and Dardas, 2015; Al-Farsi et al., 2016; Rayan and Ahmad, 2017; Amireh, 2019; Al Ansari et al., 2021; El-Monshed and Amr, 2021; Khusaifan and El Keshky, 2021). Furthermore, depression symptoms were reported among parents in nine studies (Almansour et al., 2013; Dababnah and Parish, 2013; Fido and Al Saad, 2013; Al-Farsi et al., 2016; Rayan and Ahmad, 2017; Alnazly and Abojedi, 2019; Al Ansari et al., 2021; Alshahrani and Algashmari, 2021). Anxiety was experienced by parents in six studies (Almansour et al., 2013; Al-Farsi et al., 2016; Rayan and Ahmad, 2017; Alnazly and Abojedi, 2019; Al Ansari et al., 2021). Decreased QoL of parents was found in five studies (Kheir et al., 2012; Dardas and Ahmad, 2014; Ahmad and Dardas, 2015; Alenazi et al., 2020). Physical health problems were reported in two studies (Al-qahtani, 2018; Shattnawi et al., 2021). Five studies reported social burden among parents of children with ASD (Al-qahtani, 2018; Gobrial, 2018; Alenazi et al., 2020; Al-Masa'deh et al., 2020; Shattnawi et al., 2021), while four studies reported psychological burden (Dababnah and Parish, 2013; Al-Ansari and Jahrami, 2018; Al-qahtani, 2018; Shattnawi et al., 2021). Parents of children with ASD reported financial burden in three of the studies (Dababnah and Parish, 2013; Al-qahtani, 2018; Shattnawi et al., 2021).

### Parents of Children With and Without ASD

In six studies, parents raising children with ASD were compared with parents raising children with other disabilities and children without any disabilities (Fido and Al Saad, 2013; Obeid and Daou, 2015; Al-Farsi et al., 2016; Al-Ansari and Jahrami, 2018; Amireh, 2019; Al Ansari et al., 2021). These studies were conducted in six Arab countries, namely, Bahrain, Oman, Saudi Arabia, Egypt, Kuwait, and Lebanon. The majority of these studies found that parents of children with ASD experienced higher levels of stress, reduced wellbeing, and other psychological difficulties compared to the controls. One study found that there were no significant differences between parents of children with ASD and controls in QoL or physical health (Al-Ansari and Jahrami, 2018). Furthermore, Fido and Al Saad (2013) found no significant differences in psychological distress between fathers of children with ASD and fathers of typically developing children. Only four studies made a comparison in parental stress among mothers and fathers of children with ASD. In two studies, no significant differences were found between the fathers and mothers of children with ASD in terms of depressive symptoms (Alshahrani and Algashmari, 2021), or physical, psychological, and social wellbeing (Dardas and Ahmad, 2014). However, two other studies reported that mothers experienced significantly higher levels of depressive symptoms (Al-Farsi et al., 2016) and impaired QOL in contrast to fathers (Alenazi et al., 2020).

### Factors Associated With the Impact of ASD on Parents

Factors associated with psychological disorders and the burden experienced by parents of children with ASD were explored in some of the studies. These studies found that several factors were associated with the impact of ASD on parents, including the severity of ASD, social support, economic status, maternal education, financial hardship, marital status, parental age, and the gender of the child (Al-Kandari et al., 2017; Alnazly and Abojedi, 2019; Alshekaili et al., 2019; Al-Masa'deh et al., 2020; Alshahrani and Algashmari, 2021; Khusaifan and El Keshky, 2021).

### Coping Strategies of Parents of Children With ASD

Limited studies have investigated coping strategies used by parents raising children with ASD in Arab countries. Religious coping was the most common coping strategy found in some of these studies (Dababnah and Parish, 2013; Al-Kandari et al., 2017; Amireh, 2019; Khusaifan and El Keshky, 2021). Other strategies used were acceptance, positive reframing, withdrawal from the community, denial of the ASD diagnosis, increasing social interactions, and accessing information.

### Resilience in Parents of Children With ASD

Only two studies examined the psychological resilience of parents. Dababnah and Parish (2013) reported that Palestinian parents in the West Bank demonstrated remarkable resilience in raising children with ASD. Furthermore, Alshahrani and Algashmari (2021) found high levels of resilience among parents and caregivers of children with ASD in Saudi Arabia.

## Discussion

This systematic review was conducted to identify studies that addressed the impact of ASD on parents in Arab countries. The included studies were reviewed in terms of participants, general characteristics, methodology used, and the main findings. This review found that the impact of ASD on parents and caregivers has only recently gained traction among researchers in some Arab countries. Approximately 80% of the studies included in this review were conducted in Saudi Arabia, Jordan, Egypt, Kuwait, and Bahrain. Most of these studies used cross-sectional study designs and included non-random samples. Furthermore, the study participants of the included studies comprised more mothers than fathers. Moreover, most of the included studies did not distinguish between the different subtypes of ASD.

The majority of studies found that ASD has a significant negative impact on the mental health and wellbeing of Arab parents. This finding is consistent with previous studies conducted in several countries, suggesting that parents of children with ASD experience significant levels of stress, depression, and anxiety (Loukisas and Papoudi, 2016; Reddy et al., 2019; Papadopoulos, 2021). Consistent with previous research, this review also showed that parents of children with ASD in Arab countries have poorer QoL (Lee et al., 2008; Vasilopoulou and Nisbet, 2016). Thus, parents of children with ASD face an increased risk of developing psychological disorders in contrast to parents of both typically developing children and children with other developmental disorders (Karst and Van Hecke, 2012; Schnabel et al., 2020; Papadopoulos, 2021). In addition, several studies found that these parents reported a high caregiver burden (e.g., Estes et al., 2013; Picardi et al., 2018).

Studies exploring the factors associated with stress in parents and caregivers of children with ASD are limited in Arab countries. These factors include the severity of ASD, social support, economic status, maternal education, financial hardship, marital status, parental age, and the gender of the child. Overall, these results are consistent with literature indicating a relationship between the impact of ASD on parents and various characteristics among the child and extended family (Karst and Van Hecke, 2012; Rivard et al., 2014; Iadarola et al., 2019).

Furthermore, studies addressing the coping strategies used by parents raising children with ASD are even more limited in Arab countries. Although limited, these studies found that the most common coping strategy used by parents was religious coping followed by acceptance, positive reframing, withdrawal from the community, denial of the ASD diagnosis, increasing social interactions, and accessing information. Moreover, two studies found high levels of resilience in parents and caregivers. However, since the studies are limited, these results should be interpreted with caution. Moreover, most of the studies used self-report questionnaires and lacked a qualitative measurement. As a result, socially desirable responses may have been provided by the parents and caregivers, underscoring the limitations of the data collection methods used.

The findings of this systematic review have several important implications for future research in Arab countries. Parents that have a child with ASD are severely impacted (Picardi et al., 2018). Despite the evidence provided by published studies about the impact of ASD on parents in Arab countries, there are still several knowledge gaps. For example, studies identified in this review provided minimal or no information about the impact of ASD on parental self-efficacy, physical health problems, marital relationships, or family socialization.

Furthermore, further research is required to underscore the difference in the experience of the burden associated with raising a child with ASD between mothers and fathers. While several studies have indicated that mothers of children with ASD suffer from increased levels of mental health challenges and burdens compared to fathers, some studies have yielded mixed results (Picardi et al., 2018; Al Khateeb et al., 2019; Rudelli et al., 2021). Thus, further research is required to enhance the understanding of the different challenges experienced by parents raising children with ASD in Arab countries, particularly qualitative studies (Leko et al., 2021).

Moreover, the majority of the studies included in this review used small non-random samples based on a limited number of countries. Consequently, this limited the generalizability of the findings to all parents in the Arab region. Thus, future research including larger random samples is required.

### Limitations

Several limitations should be considered when interpreting the findings of the current review. First, only studies published in English in peer-reviewed journals were included in this review. The researchers excluded literature published in the Arabic language due to the severe lack of Arabic electronic databases. Furthermore, conducting a comprehensive manual search would have been a formidable task (Alkhateeb and Alhadidi, 2019). As a result, the included studies may not be representative of all the evidence (Morrison et al., 2012). Second, narrow search parameters were applied resulting in the exclusion of other potential sources of information such as conference papers and theses or dissertations that could uncover other relevant literature. Third, this review did not include a critical appraisal of the identified studies. Although the review was confined to studies published in peer-reviewed journals (71% of which had an Impact Factor >1.0), this does not necessarily guarantee the methodological quality of reviewed studies. Therefore, some of the included studies may be of low quality, warranting further research. Despite these limitations, this systematic review facilitates the understanding of the impact of ASD on parents in Arab countries.

## Conclusion

This systematic review summarized the results of studies underscoring the impact of ASD on parents in Arab countries. Twenty-four studies met inclusion criteria and most of the included studies were quantitative studies that were conducted within the last 5 years in Saudi Arabia, Jordan, Egypt, Kuwait, and Bahrain. Furthermore, most of the identified studies found that ASD has a significant negative impact on the mental health and wellbeing of Arab parents. Moreover, significant gaps were found in the evidence base, including research on coping strategies and interventions aimed at reducing stress and burden among parents and caregivers. However, this review contributes valuable insights for future studies on parents of children with ASD in Arab countries.

## Data Availability Statement

The original contributions presented in the study are included in the article/supplementary material, further inquiries can be directed to the corresponding author/s.

## Author Contributions

JA conceptualized the manuscript and research questions, performed the initial article search, and reviewed all articles identified during the search. MH and WM participated in the acquisition of data, analysis, and manuscript drafting. All authors read and approved the final manuscript.

## Conflict of Interest

The authors declare that the research was conducted in the absence of any commercial or financial relationships that could be construed as a potential conflict of interest.

## Publisher's Note

All claims expressed in this article are solely those of the authors and do not necessarily represent those of their affiliated organizations, or those of the publisher, the editors and the reviewers. Any product that may be evaluated in this article, or claim that may be made by its manufacturer, is not guaranteed or endorsed by the publisher.
